# The Keystone Flap: A Game Changer That Promises New Horizons in Reconstructive Surgery

**DOI:** 10.7759/cureus.69297

**Published:** 2024-09-12

**Authors:** Anca Bordianu, Ion Petre, Catalin Bejinariu

**Affiliations:** 1 Plastic and Reconstructive Department, "Bagdasar Arseni" Emergency Hospital, University of Medicine and Pharmacy "Carol Davila", Bucharest, ROU; 2 Functional Science, Medical Informatics, and Biostatistics, Victor Babes University of Medicine and Pharmacy Timisoara, Timisoara, ROU; 3 Plastic and Reconstructive Surgery, "Bagdasar Arseni" Emergency Hospital, Bucharest, ROU

**Keywords:** trauma, local flaps, perforators, reconstructive surgery, keystone flap

## Abstract

Background: Since its first description emerged in 2003, the keystone flap has garnered the attention of the international scientific community due to its high safety profile and the suitably low complication rate associated with the reconstructive process.

Materials and methods: In this study, data were obtained from the performance of 72 keystone flaps to cover soft-tissue defects after the excision of neoplastic processes, excisions, and injuries occurring in polytrauma. The study was conducted in the Department of Plastic Surgery, "Bagdasar-Arseni" Emergency Hospital, Bucharest, and two plastic surgeons in the department performed the surgical procedures.

Results: The statistical analysis revealed a remarkably low complication rate (3.22%), excellent functional and esthetic results, and a short hospitalization time. No intraoperative complications were identified during this study. The degree of satisfaction obtained after reconstructive surgery was exceptionally high, with a score of 9.47 on a 10-point rating scale (0 = poor results, 10 = excellent results) from the patient's perspective and 9.51 out of 10 for the surgical team.

Conclusions: The keystone flap is the optimal solution for reconstructing soft-tissue defects of variable sizes and shapes. It is associated with a low length of hospitalization, a low complication rate, and high patient satisfaction.

## Introduction

The definition of angiosomes in the early 1970s opened new horizons in identifying reconstructive methods adapted to the specificities and particularities of different soft-tissue defects [1]. The international scientific community then became interested in the applicability of the new concept to study the reliability of the newly identified reconstructive methods and establish their safety profile [2-4].

Further research created the context for Byun and coworkers' first description of the keystone flap concept in 2003 [5]. The new reconstructive technique offered significant benefits in covering medium and large soft-part defects, maintaining local morphologic features, and increasing the safety profile. The complication rate associated with this reconstructive method was significantly reduced compared to similar reconstructive techniques [6-9].

The name of this flap is derived from the association of its shape and function with the central keystone at the top of Roman arches, which provides strength to and holds together the whole structure. Similarly, by covering significant defects, the flap offers an optimal solution regarding the structural strength of the reconstructed anatomic regions [10-12].

As described by Behan [10], the keystone flap is a trapezoid-shaped local flap, whose vascularization may be random or based on perforating arteries and which can be used alone or in combination with a similar contralateral flap to cover defects of variable dimensions [13-17]. In its classic form, this reconstructive method involves the realization of a drape-like flap. The width of this flap should be equal to that of the soft-tissue defect to be covered, with the sides of the trapezium being drawn at angles of 90°/90° to the proximal tangent of the defect [18].

## Materials and methods

The study was conducted in the Department of Plastic Surgery, "Bagdasar-Arseni" Emergency Hospital, Bucharest, and two plastic surgeons in the department performed the surgical procedures. "Bagdasar-Arseni" Emergency Hospital Ethics Commitee issued approval (no. 26290).

This study analyzed the evolution of 72 classic keystone flaps, either simple or combined, in the case of significant defects. To conduct the research, the data collected included the following parameters: demographic data, duration of surgery, length of hospitalization, rate and type of complications, patients' satisfaction with the outcome, and analysis of the degree of surgery complexity from the surgeon's perspective. Microsoft Excel (Microsoft Corp., USA) was used to perform the statistical analysis.

Inclusion criteria

Male and female patients, aged 18-65 years and hospitalized for the treatment of tumor pathology, decubitus ulcers, and posttraumatic soft-tissue defects, were included in the study.

Exclusion criteria

Patients with severe cardiac disease (New York Heart Association (NYHA) III heart failure classification stage), arteriopathy, psychopathy, and poorly controlled diabetes mellitus were deemed ineligible to participate in this study.

Flap planning and design

The preoperative drawing was conceived in close accordance with the nature of the pathology identified. In the case of oncologic diseases, the remaining defect after excision respected the oncologic safety margins, and the objectification of the radicality of excision was supported by the extemporaneous histopathological examination performed. In posttraumatic soft-tissue defects, the size of the defect was assumed to increase by 3-5 mm after the excision of the wound edges.

The first step in the preoperative design was to create a perilesional ellipse. Then, the lateral edges of the flap were drawn at angles of 90°/90° to the proximal tangent, and a semicircular line (the distal edge of the flap) was joined to the flap edges so that the flap's width equaled that of the defect to be covered.

Surgical technique

The first stage of the flap dissection was represented by the realization of the integumentary incision according to the preoperative marking, respecting the anatomical plans and vascular structures at this level. The depth of the dissection was dictated by the local particularities of the targeted anatomical region, with sectioning of the superficial muscle fascia to facilitate the mobilization of the flap in all cases that allowed it.

**Figure 1:**
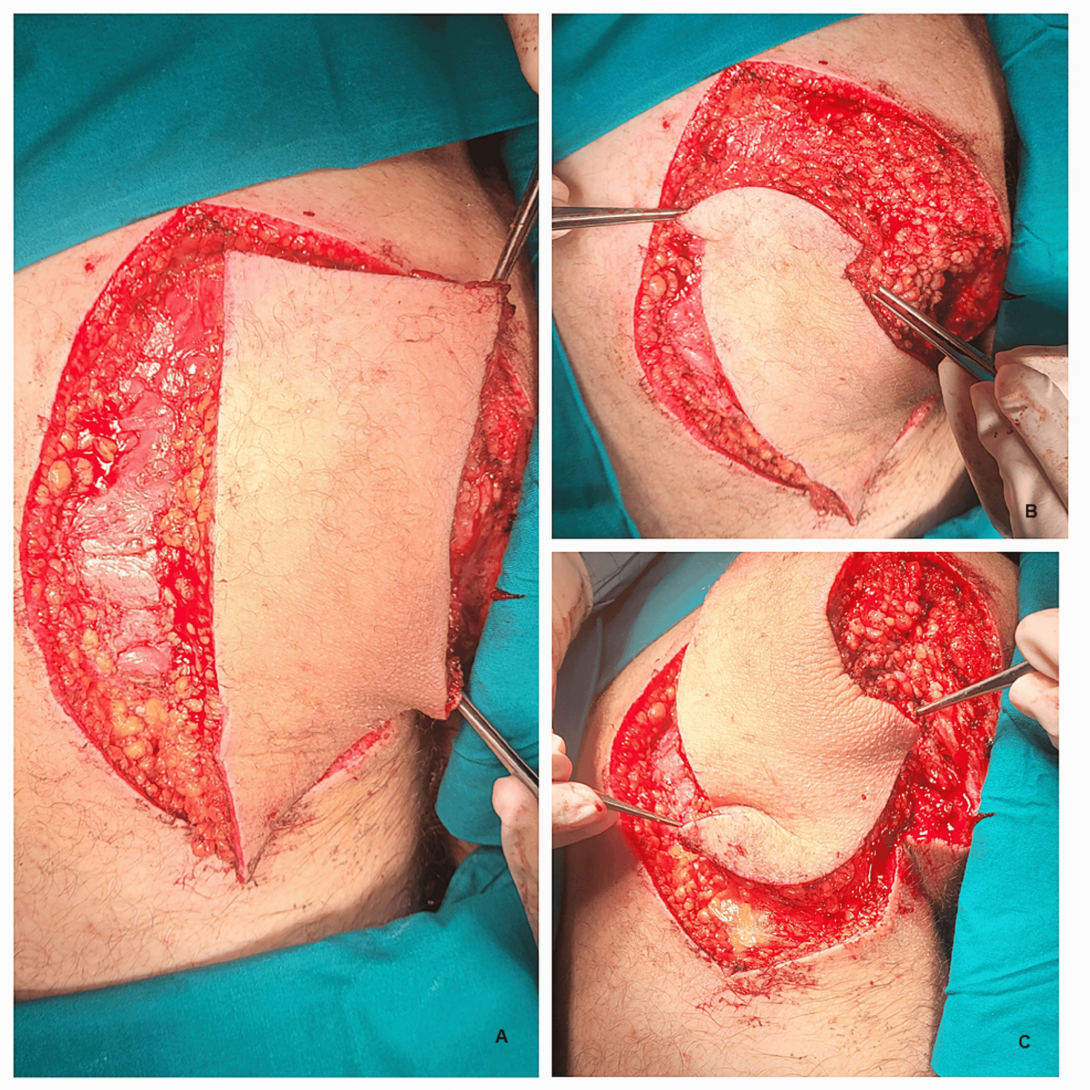
Intraoperative photographs showing the circumferential incision of the fascia: A and B) Flap incision; C) Flap mobilization.

As previously mentioned, a high degree of mobility can be obtained with circumferential sectioning of the fascia in the deep plane of the flap. Mobilization is limited without sectioning, which is significantly challenging when covering the soft-tissue defect. Whenever possible, the option of sectioning the superficial muscle fascia outside the projection area of the flap was chosen so that after its retraction, the surface area of the fascia was equal to the flap's surface. In practice, the authors sectioned the superficial muscle fascia at 1 cm from the projection area of the flap (as shown in Figure 1).

**Figure 2:**
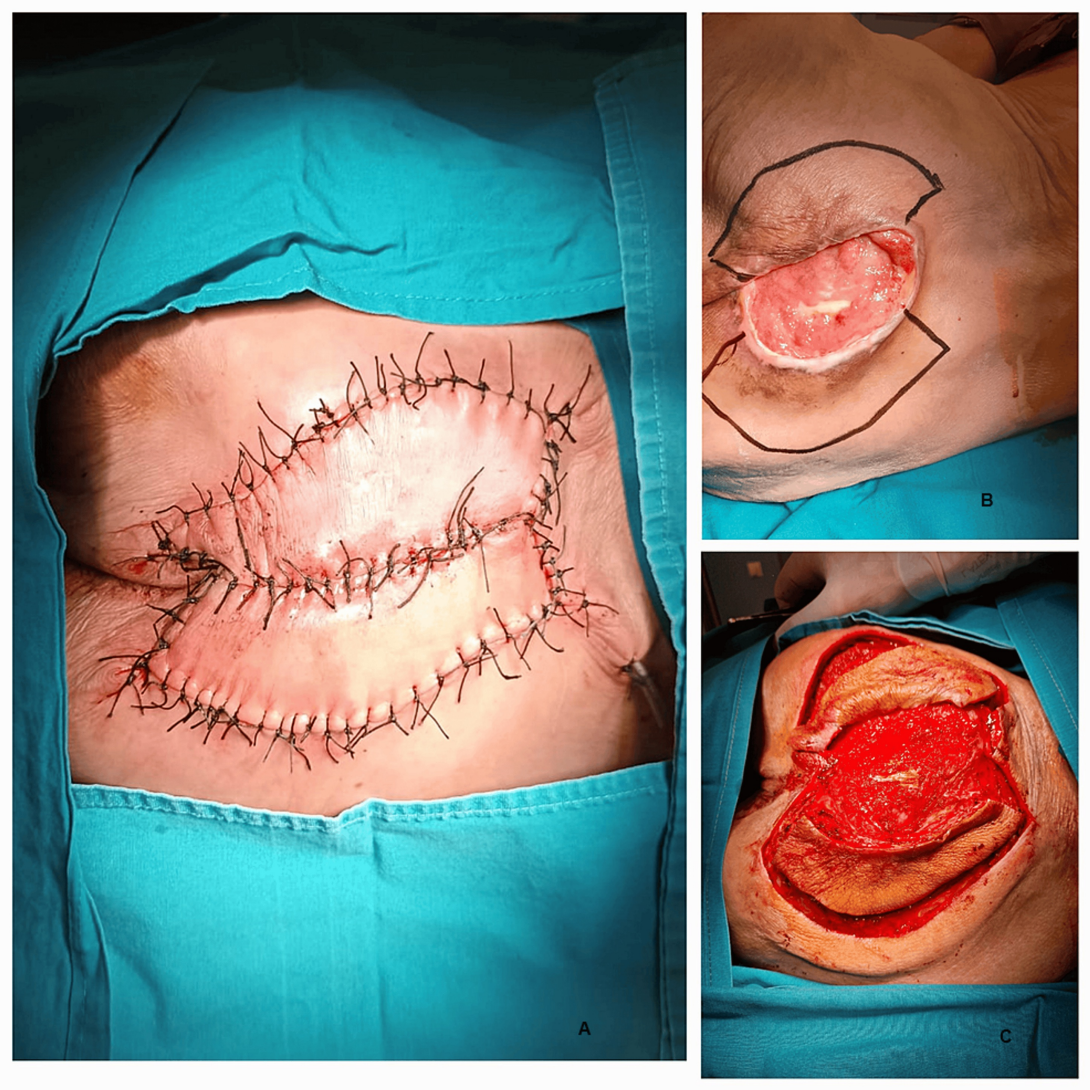
Main steps in the flap dissection and mobilization: A) Keystone flap completed; B) Flap design and the defect; C) Flap's mobilization.

To increase the flap's mobility and reduce tension at the suture level, the dissection was performed deep within the flap, without exceeding 5% of its width and with great care to avoid severing the perforating arteries at this level (as shown in Figure 2).

The degree of satisfaction with the outcome of the reconstructive surgical procedure was evaluated through a questionnaire in which both patients and the operating team expressed their opinions by assigning scores ranging from 1 to 10.

## Results

This research analyzed the evolution of 72 keystone flaps. The patients' ages ranged from 32 to 64 years, with a mean of 41.35 years and a female-to-male ratio of 3:1.

Regarding the nature of the soft-tissue defect that required reconstruction, 38% of the cases were post-excisional defects due to oncologic reasons, 34% were decubitus ulcers, and 28% were posttraumatic defects.

**Figure 3:**
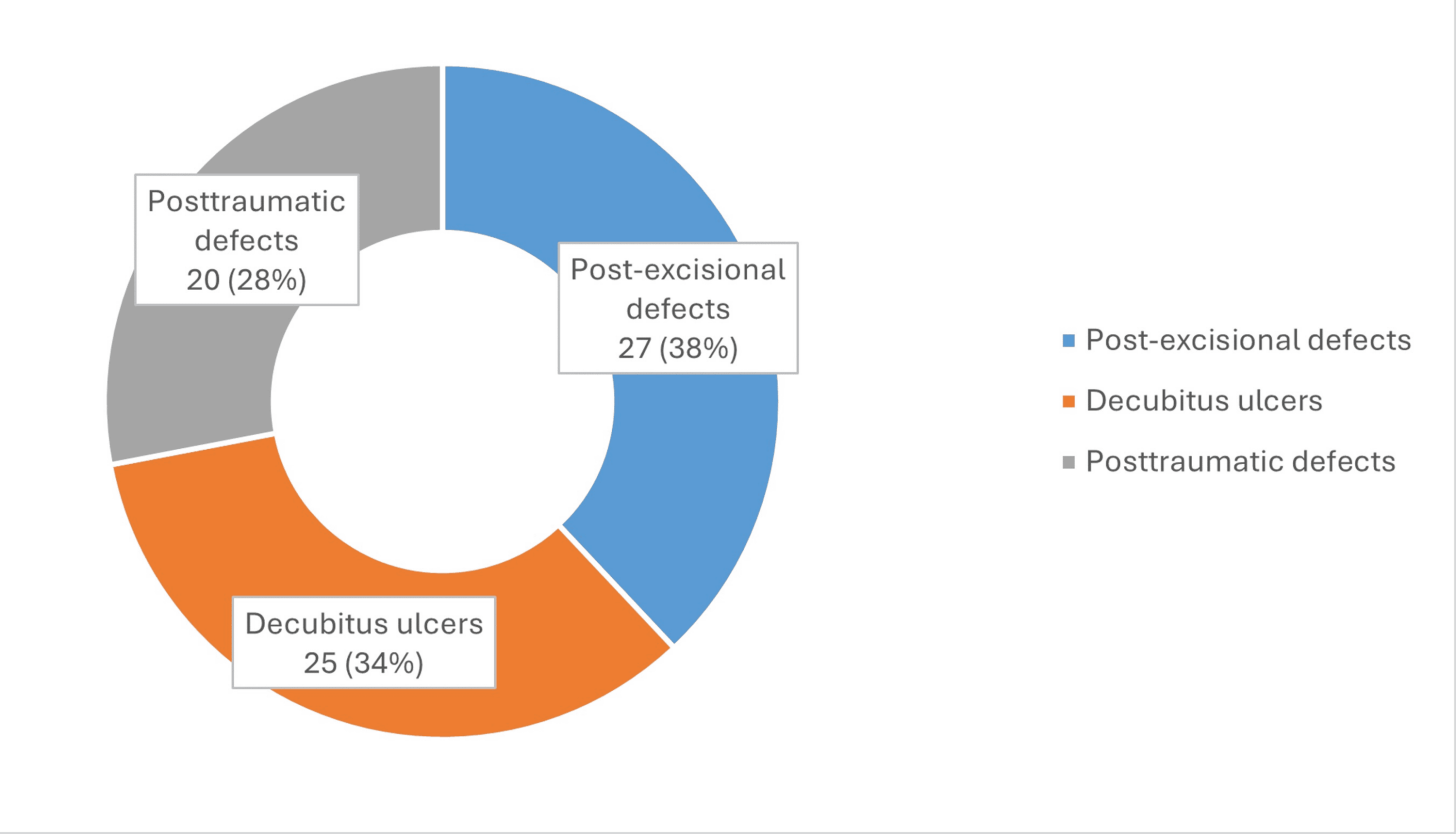
Types of defects in the lot.

**Figure 4:**
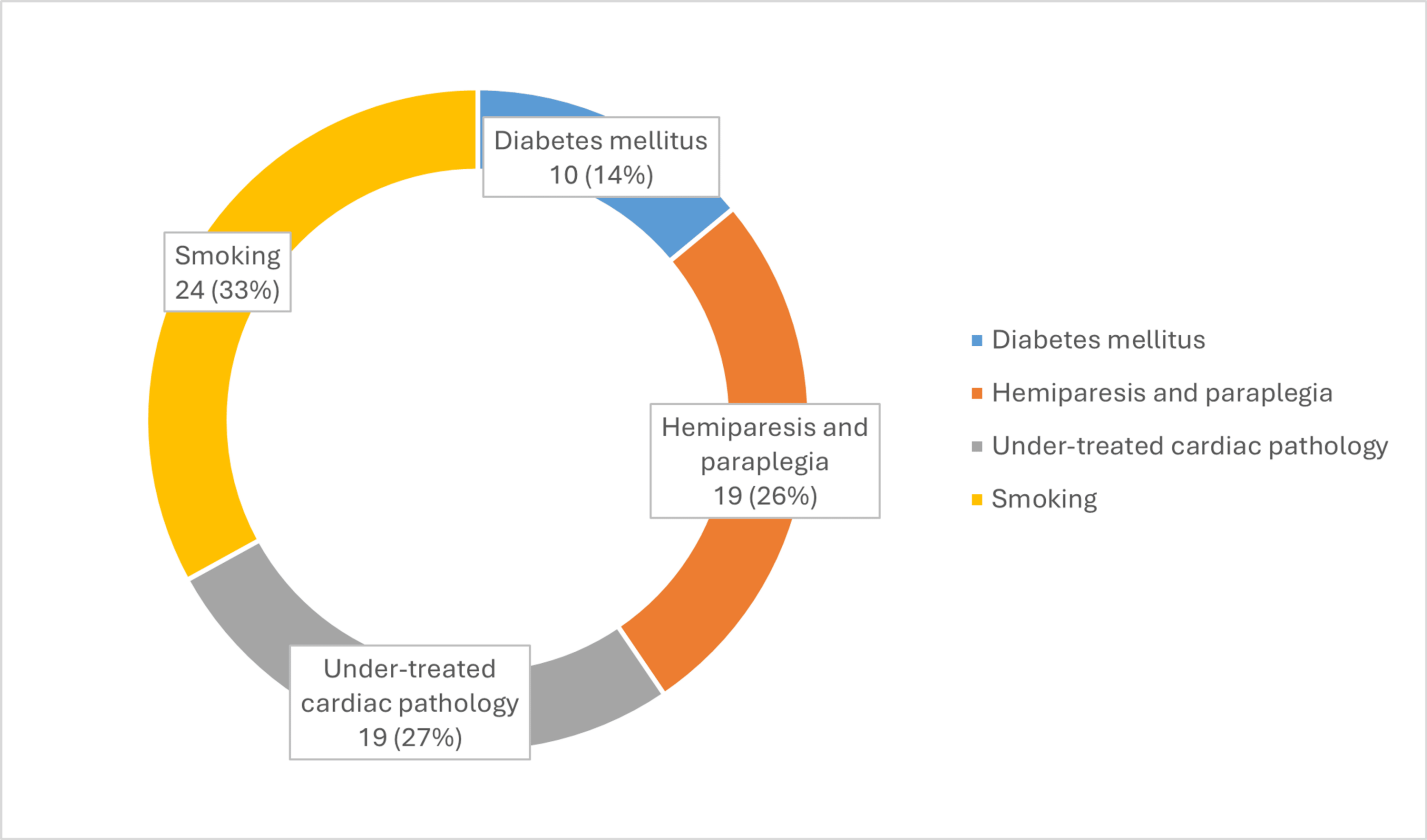
Comorbidities associated with the lot.

The risk factors identified in the group of patients who underwent the reconstructive protocol included smoking (more than 20 cigarettes per day) in 33%, undertreated cardiac pathology in 27%, hemiparesis and paraplegia in 26%, and diabetes mellitus in 14% (as represented in Figures 3-4).

The postoperative complication rate was 3.22%, with two cases identified in which reintervention was required to correct midline dehiscence (maximum tension line). Both cases were identified in patients immobilized in bed due to associated neurological lesions.

**Figure 5:**
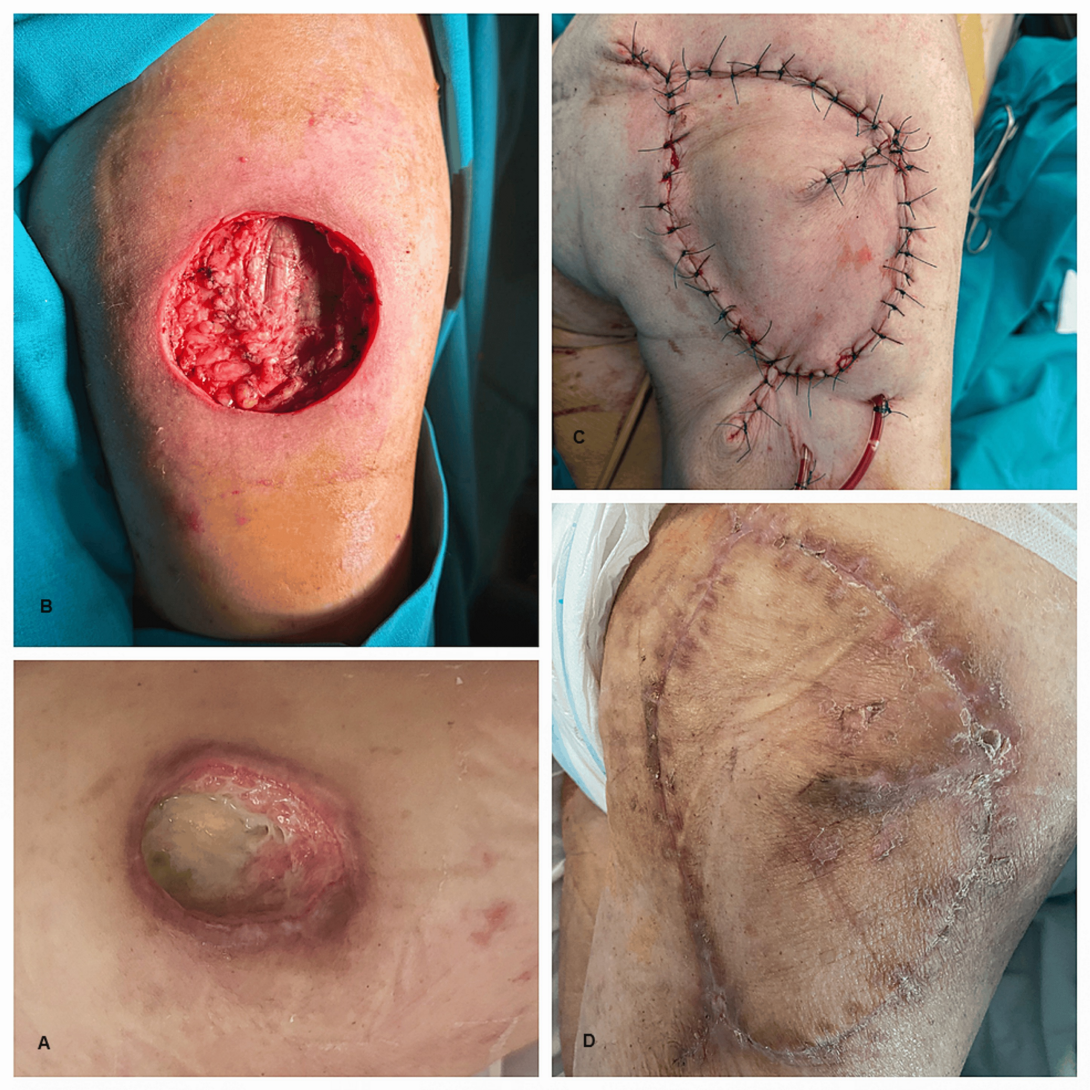
An 81-year-old patient hospitalized for the surgical treatment of the right trochanteric pressure sore (images show the preoperative appearance, the soft-tissue defect that remained after excision, the keystone flap in position, and the postoperative aspect on day 14): A) Trochanteric eschar; B) Defect after debridement; C) Keystone flap completed from the lateral aspect of the thigh.

The mean length of hospitalization was 5.7 days, significantly influenced by the hemiplegic and paraplegic patients included in the study, who remained hospitalized for up to 14 days due to difficult mobilization (Figure 5). The suction drain was removed when its volume fell below 5 ml every 24 hours. Further, the sutures were removed 14 days postoperatively.

**Figure 6:**
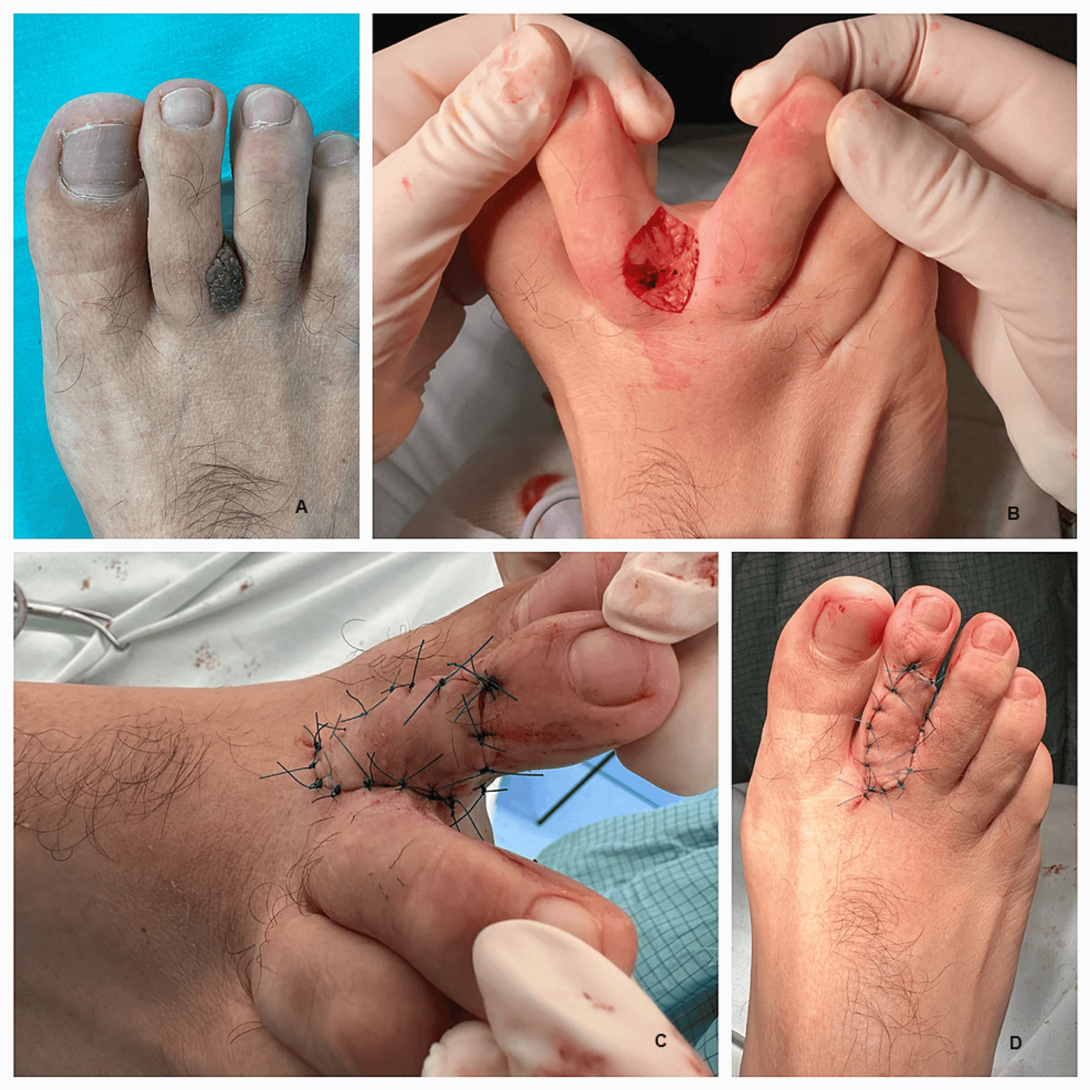
A 45-year-old patient admitted for the treatment of a benign tumor located on the second finger (images show the preoperative appearance, the soft-tissue post-excisional defect, and the keystone flap in position): A) Benign tumor between toes; B) Defect after excision; C and D) Keystone flap completed.

The keystone flap was used to reconstruct soft-tissue defects of the foot in carefully selected cases (Figure 6).

**Figure 7:**
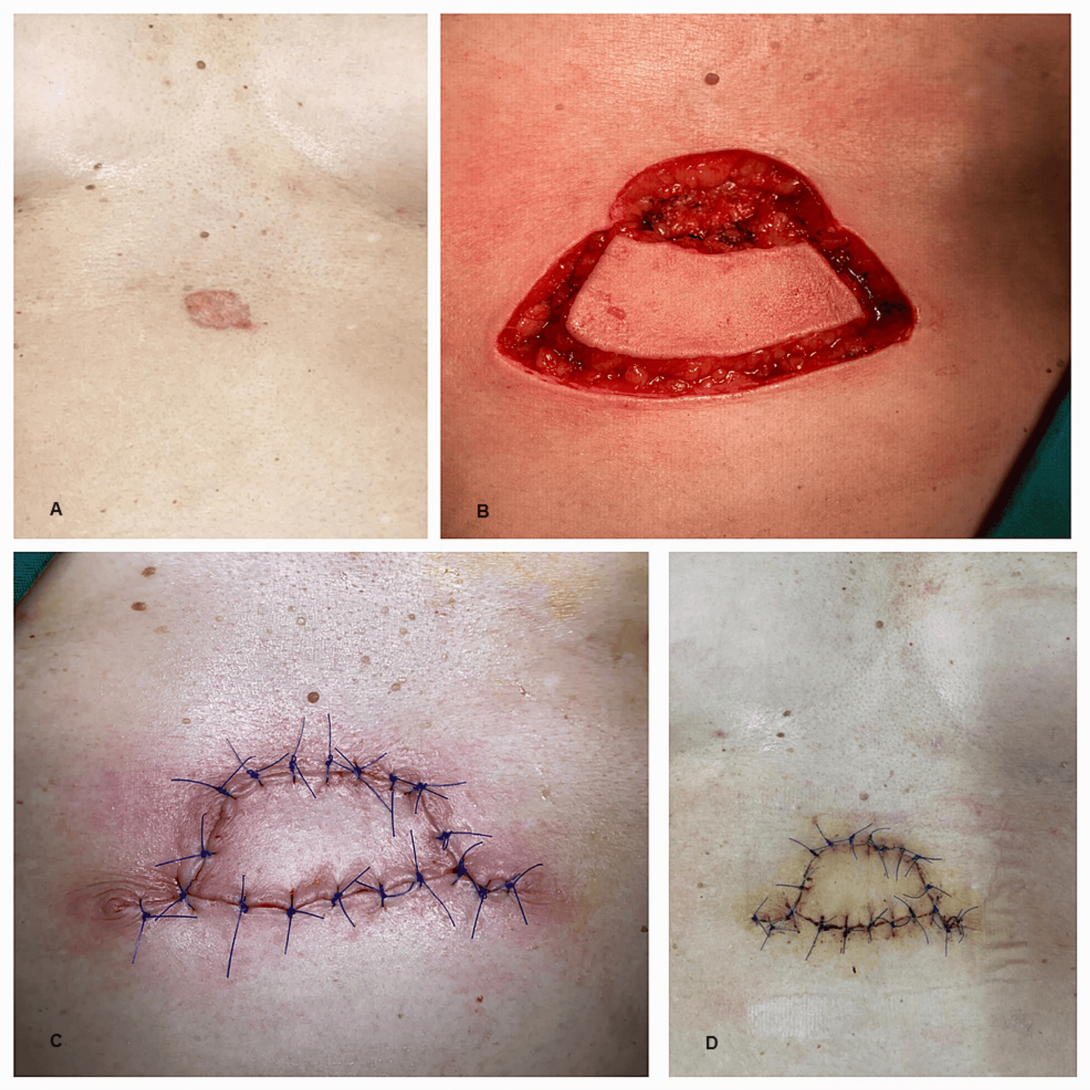
A 46-year-old patient hospitalized for the treatment of basal cell carcinoma of the epigastric region (images show the preoperative appearance, post-excision soft-tissue defect, keystone flap dissection, and postoperative appearance): A) Skin tumor on the epigastric region; B) Keystone flap dissection; C and D) Keystone flap completed.

The rich vascularization, increased tissue mobility, and well-represented adipose tissue at this level contributed to excellent functional and esthetic results (Figure 7).

**Figure 8:**
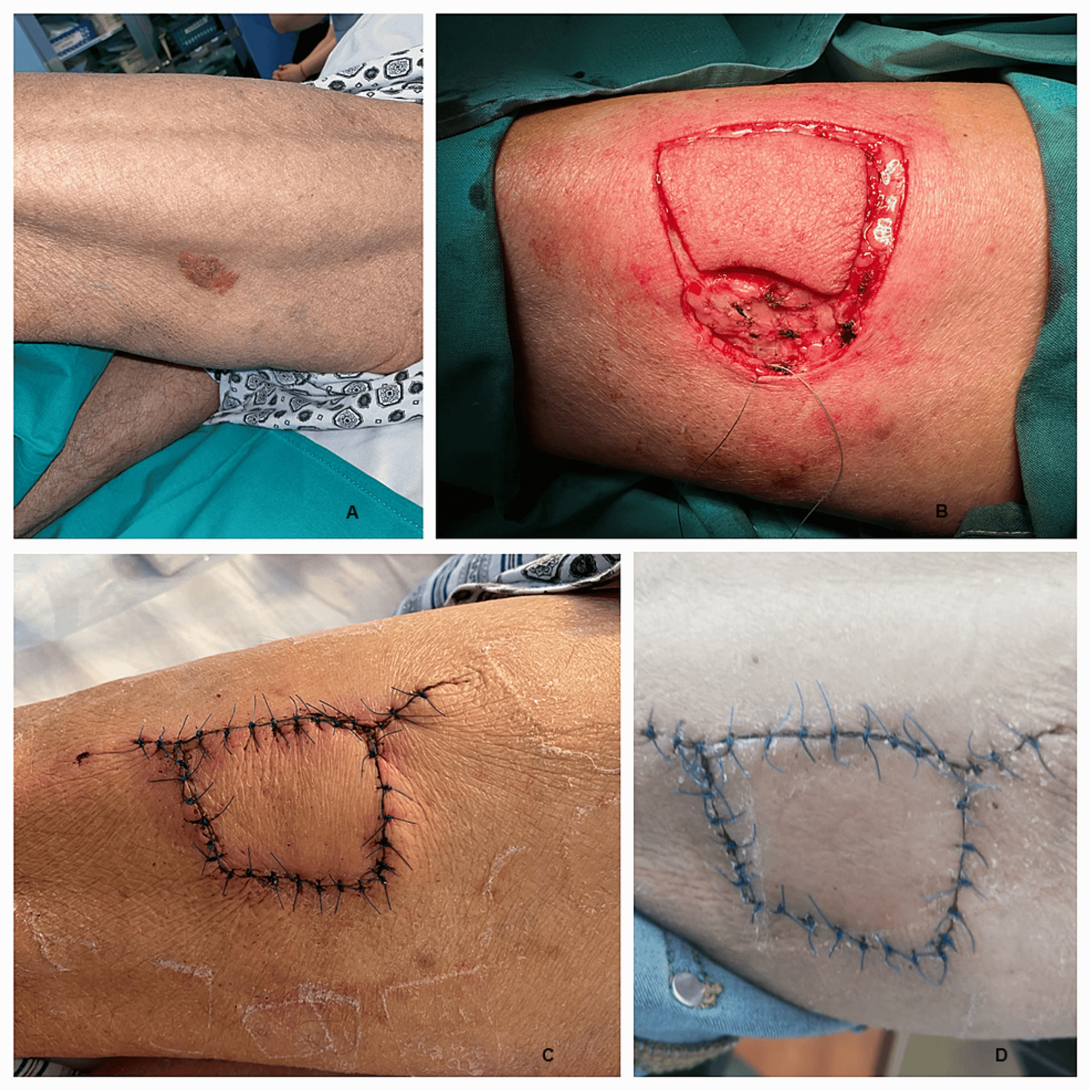
A 53-year-old patient hospitalized for the treatment of a left thigh tumor (images show the preoperative aspect, the dissected keystone flap, and the postoperative appearance: A) Skin tumor on the thigh; B) Keystone flap dissection; C and D) Keystone flap completed.

The flexibility of the thigh tissue offered encouraging prospects for using this type of flap to cover the remaining defects of trauma or tumors (Figure 8).

**Figure 9:**
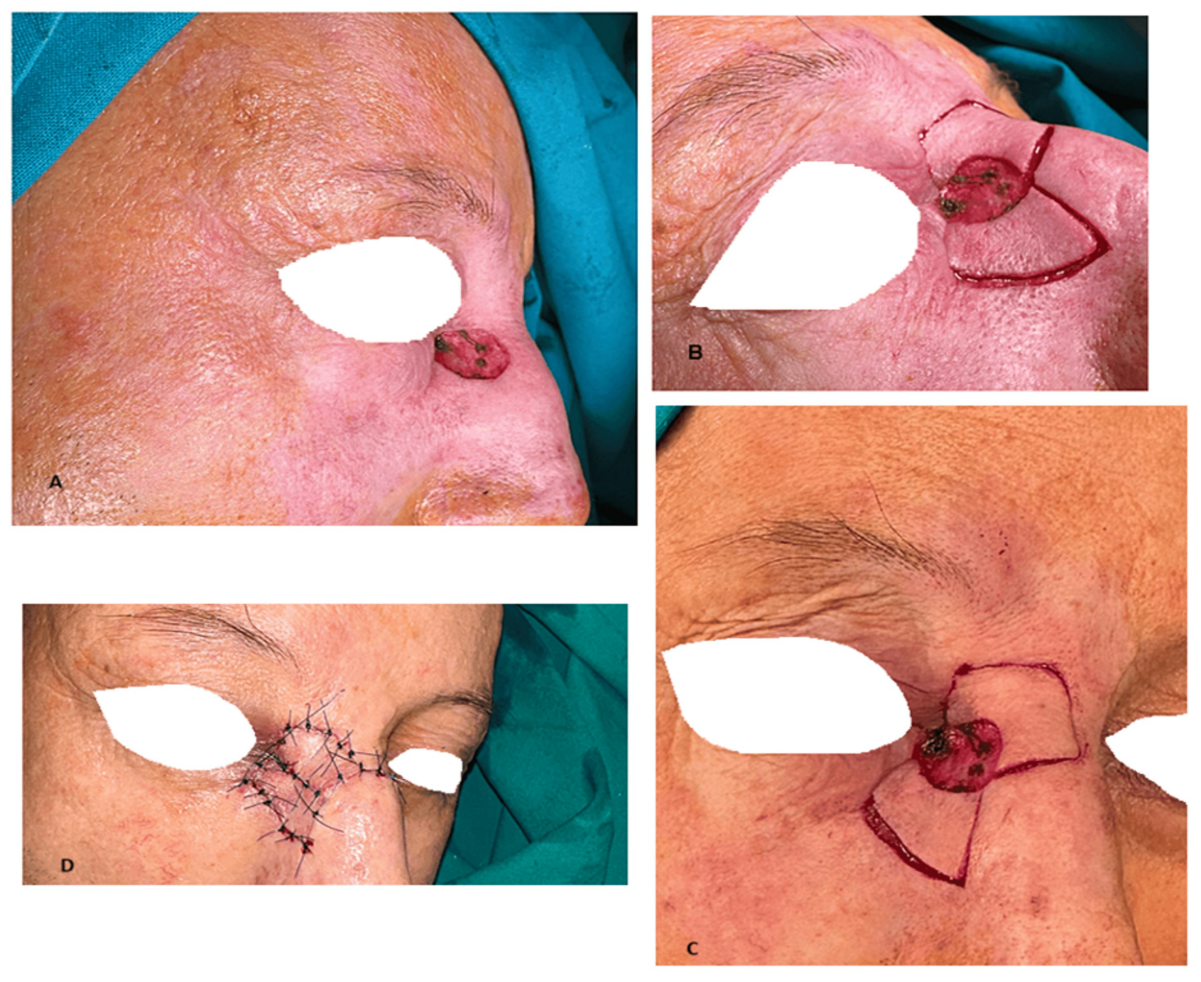
A 48-year-old patient admitted for the treatment of a nasal tumor (images show the soft-tissue defect, symmetrical keystone flaps, and the postoperative appearance): A) Defect after skin tumor removal; B and C) Different aspects of the bilateral keystone flap dissection; D) Bilateral keystone flap completed.

The keystone flap was also used for soft-tissue defects in the facial region, providing satisfactory results both volumetrically and esthetically, with the association of symmetrical flaps offering impressive three-dimensional perspectives (Figure 9).

**Figure 10:**
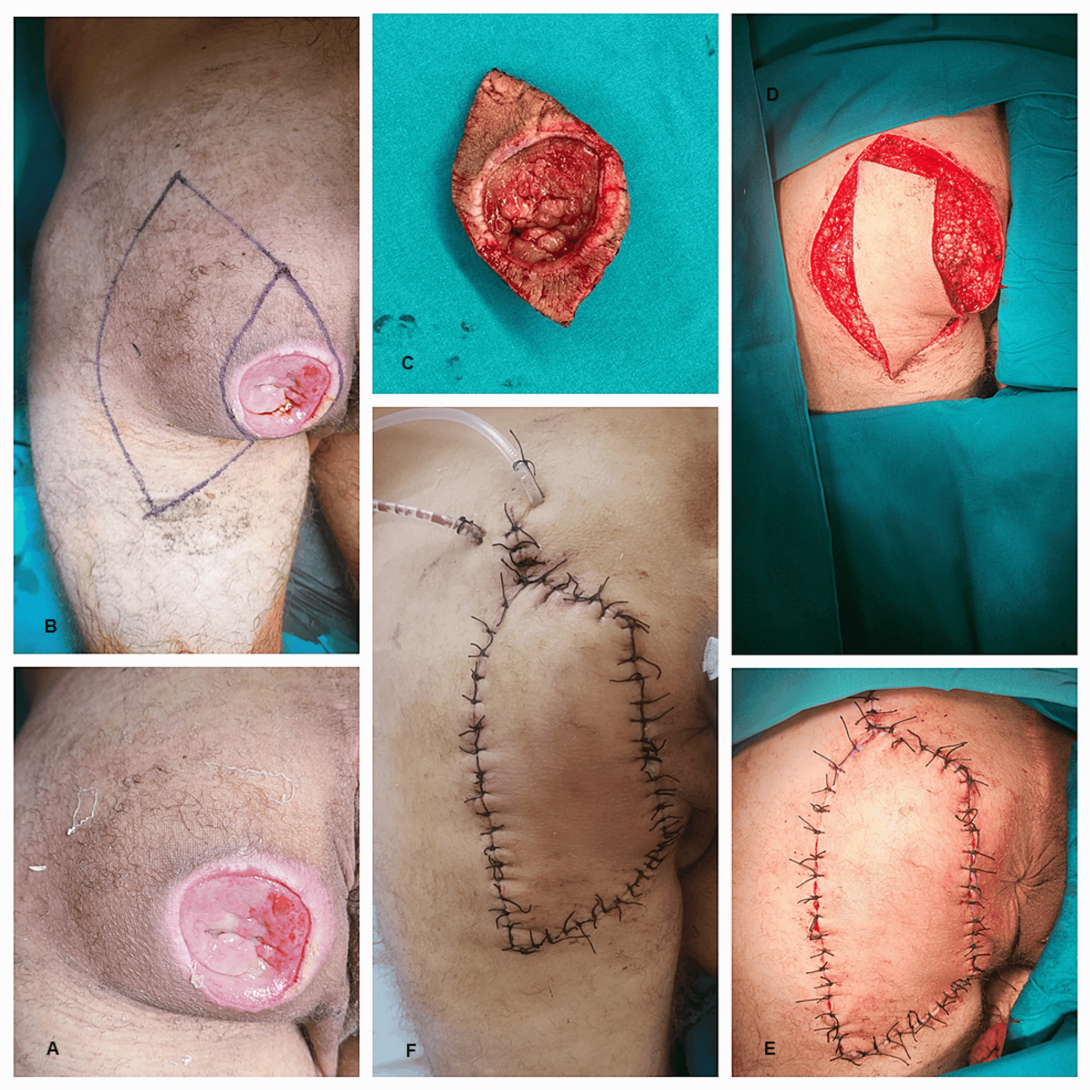
Steps of keystone flap reconstruction in a patient with a gluteal decubitus ulcer: A) Decubitus ulcer; B) Keystone flap design; C) Excised part; D) Keystone flap dissection; E and F) Keystone flap completed.

The keystone flap has also proven helpful in treating decubitus ulcers, particularly in patients who can be mobilized and whose injuries have occurred after being discharged following long periods in intensive care units. In these cases, covering soft-tissue defects with fasciocutaneous flaps was the optimal solution, as the contribution of the muscle mass characteristic of myocutaneous flaps is unnecessary (Figure 10).

The overall patient outcome was favorable, characterized by rapid flap integration and high patient satisfaction.

## Discussion

In its various forms, the keystone flap is a life-saving reconstructive technique in most situations requiring the coverage of variable dimensions of soft-tissue defects [19]. Used for reconstructing small-sized defects in the facial region, these flaps offer the advantage of preserving the particularities of the local morphology without unsightly scarring in the recipient area and with minimal distortion of the regional anatomy [20-24].

Large flaps can cover defects caused by neoplastic processes, giant congenital nevi, and trauma, which result in soft-tissue defects [25].

Depending on the particularities of the case, the size of the soft-tissue defect, and the pathology that required reconstruction, keystone flaps can be adapted to meet all the requirements of the reconstructive protocol [26,27]. In this regard, several types of flaps have been described and differentiated according to the criteria related to the specificity of the dissection, its depth, and the number of flaps used (Table 1).

**Table 1:** Keystone flap classification.

Keystone flap	Dissection particularities
Type I	Classic flap—without deep fascia dissection
Type II	Sectioning of the deep fascia at the level of the convex edge of the flap
Type III	Symmetrical flaps for covering large defects
Type IV	Undermined flaps up to two-thirds of their surface

Although keystone flaps prove to be helpful in most reconstructive surgeries, special attention should be paid to anatomic regions where the low skin elasticity makes mobilizing the flaps and suture postoperative wounds challenging. In this regard, the authors have identified the following areas at high risk of complications: the knee region, the distal one-third segment of the calf, and the leg [28,29]. However, they have demonstrated that the keystone flap can be used to reconstruct soft-tissue defects of the foot in carefully selected cases.

The literature and practical experience of the authors confirm that keystone flaps are the optimal solution for the coverage of medium and large soft-tissue defects of the trunk, buttocks, and thigh. This reconstructive technique has also provided good functional and esthetic results in facial reconstructive surgery. The absence of a secondary donor area, the preservation of the morphologic particularities of the replaced tissue, and the lack of the volumetric deficit characteristic of this reconstructive technique make it a feasible method to consider in all cases requiring coverage of soft-tissue defects. Moreover, the keystone flap is the optimal reconstruction solution for soft-tissue defects in the torso and abdominal regions. Its rich vascularization, increased tissue mobility, and well-represented adipose tissue at this level contribute to excellent functional and esthetic results.

Although the keystone flap is the optimal solution in most situations requiring coverage of large soft-tissue defects, more attention should be paid to posttraumatic injuries. Detachment of the structures adjacent to the defect during trauma may have adverse effects on the viability of the flap and on the healing period after the reconstructive process [30].

The keystone flap proves its efficiency in covering soft-tissue defects localized in various anatomical regions. The flexibility of thigh tissue offers encouraging prospects for using this type of flap to cover defects remaining after trauma or tumors.

Another aspect to consider when performing reconstruction is careful dissection to preserve the perforator arteries, as their damage may be associated with marginal necrosis followed by postoperative wound dehiscence.

The keystone flap can also be used for soft-tissue defects in the facial region. It offers satisfactory results from a volumetric and esthetic point of view, and the association of symmetrical flaps offers impressive three-dimensional perspectives. It has also proven helpful in treating decubitus ulcers, where covering soft-tissue defects with fasciocutaneous flaps is the optimal solution.

The rate of complications associated with this reconstructive technique is low, with the present study identifying only two cases of wound dehiscence. In the context of bed immobilization, this complication can be justified by decubitus formation in the operated area since no marginal necrosis or signs of local inflammation were identified.

In terms of patient satisfaction with the outcome and overall experience of the therapeutic protocol, the short hospitalization time, the low complication rate, and the good esthetic outcome contributed significantly to the high value of this indicator.

From the surgical team's perspective, the reconstructive protocol requires a low learning curve. There is no need for microsurgical skills or specific competencies that would make it difficult to assimilate the information needed to perform the procedure safely. In addition, no specific instrumentation is required to perform reconstructions using the keystone blade. Therefore, the costs associated with surgery do not exceed those of classic operations.

Although the keystone flap proves its efficiency in many situations, its use is limited in Romanian hospitals. The factors that could be behind this phenomenon could be the relatively low number of scientific materials that provide information about the reconstructive technique and the lack of practical experience confirming the effectiveness of this technique. Therefore, the authors believe that publishing scientific articles on the reconstructive protocol, its indications, and results may be crucial in establishing the keystone flap as the gold standard for most locoregional flap reconstructions.

Regarding the learning curve of the reconstructive technique using the keystone flap, the authors noted that the teams of resident physicians who dealt with the cases included in the study could perform the surgery without technical problems, starting with the third operation. Therefore, this reconstructive technique has undeniable advantages.

As for the duration of surgical interventions, it should be noted that the interval required for flap dissection is between 30 minutes and 60 minutes, depending on the flap's dimensions and the subtype used. The prolonged duration is characteristic of type IV (as per Table 1), in which the meticulous dissection of the deep plane is time-consuming.

When comparing our results with those of other studies regarding the keystone flap, the authors remarked that their current results are comparable to previous findings. Due to trauma, the use of the keystone flap is limited by the absence of perforators at the donor site. Additionally, the defect's transverse alignment impairs tissue mobility, due to which the flap cannot be advanced. Similarly, defects affecting tissues with less mobility can be challenging. The keystone flap is currently underused due to its reconstructive potential. Studies and information dissemination may be effective ways of popularizing this reconstructive technique.

## Conclusions

The keystone flap is a life-saving method for soft-tissue defects of variable sizes remaining after the excision of neoplastic processes, decubitus eschar, and skin defects resulting from polytrauma. It can be used as a first-line treatment for most soft-tissue defects and as a backup solution in cases where microvascular reconstructive methods do not provide the expected results. With its high safety profile, low operative time, low complication rate, and reduced length of hospitalization, this reconstructive technique is associated with increased patient satisfaction with the therapeutic protocol.
